# Auditory enrichment on facial and physiological responses of Pantaneiro geldings and mares under short-term stress

**DOI:** 10.1371/journal.pone.0323649

**Published:** 2025-05-20

**Authors:** Vanessa Cristini Sebastião da Fé, Viviane Maria Oliveira dos Santos, Ana Caroline Bini de Lima, Maria Simara Palermo Hernandes, Fabiana Ribeiro Caldara, Marina De Nadai Bonin Gomes

**Affiliations:** 1 Graduate Program in Animal Science, Faculty of Veterinary Medicine and Animal Science, Federal University of Mato Grosso do Sul, Campo Grande, Brazil; 2 Graduate Program in Animal Science, Faculty of Agricultural Sciences, Federal University of Grande Dourados, Dourados, Brazil; Universidade Federal de Mato Grosso do Sul, BRAZIL

## Abstract

This study aimed to evaluate the effect of auditory stimuli on mares and geldings during social isolation and movement restriction, and their ability to promote the reduction of stress responses. The research was conducted with eight Pantaneiro horses, divided into Experiment I (4 mares) and Experiment II (4 geldings), both experiments were executed equally using a 4x4 Latin square design with experimental (classical, country and new age) and control (no music) treatments. Physiological parameters (heart rate, heart rate variability, respiratory rate, ocular temperature by infrared thermography, and surface temperature by infrared thermometer) and facial expressions (*eye closure* (AU143), *blink* (AU145), *half blink* (AU47), *inner brow raiser* (AU101), *eye white increase* (AD1), *tongue show* (AD19), *nostril dilator* (AD38), *chewing* (AD81), *ears forward* (EAD101), and *ear rotator* (EAD104)) were assessed throughout the 24 minutes that the animals remained in the restraining stock. In Experiment I, there was a reduction (p < 0.05) in heart rate during classical and country treatments and an increase (p < 0.05) in auricular and body temperatures during the classical treatment. Additionally, the frequencies of expressions of *nostril dilator* (AD38), *ear rotator* (EAD104), and *half blink* (AU47) were lower (p < 0.05). In Experiment II, there was an increase (p < 0.05) in ocular temperature during the country and control treatments, and a higher frequency (p < 0.05) of *ears forward* (EAD101) during the country treatment. Exposure to Beethoven’s 9th Symphony (classical genre) and Hank Williams Jr.’s “Ramblin’ In My Shoes” (country genre) reduced stress in mares. Geldings showed less pronounced responses to music genres, indicating a possible preference for Janet Marlow’s “Horsing Around” (new age genre). Music can be used as auditory enrichment for horses in the specific context of this study.

## Introduction

The Pantaneiro horse originated from Iberian horses brought to Brazil during the colonial period [1]. Once introduced into the Pantanal, these horses underwent natural selection over the years, developing characteristics that form an ecotype well-adapted to the environmental conditions of this biome, characterized by high temperatures, and periods of flooding and drought [2,3]. The breed is highly relevant to the local population, serving as an important means of transportation, particularly in regions with difficult access. Additionally, the notable adaptive traits of the Pantaneiro horse confer unique genetic value and significant utility in cattle management, which is the main economic activity in the region [4]. Currently, there is a growing interest in the husbandry of this native breed.

Equines, like other domesticated species, have basic physiological and behavioral needs that require attention. When the basic needs of the species are not met, welfare is compromised [5]. Thus, the domestic environment can be extremely challenging, particularly for animals kept under suboptimal conditions, as is the case in intensive farming systems where the expression of natural behavior is restricted [6].

It is common for stabled horses to be kept in social isolation. However, for a species that naturally lives in herds, close contact with conspecifics is ideal due to evolutionary pressures and their vulnerability as prey [7]. In this context, the restriction of movement resulting from the physical limitations imposed on animals raised in intensive systems is extremely detrimental, considering that free-ranging horses travel several kilometers daily [8]. In addition to social isolation and movement restriction, the routine management performed using restraining stocks, whether for medical intervention, sanitary control, or reproductive purposes, can induce stress [9], and consequently, have a negative impact on welfare.

In this scenario, auditory environmental enrichment emerges as a means to enhance the quality of life and ensure the welfare of equines by reproducing sound for animals that do not have access to open environments or have been temporarily isolated. It can also be used when introducing an animal to an unfamiliar enclosure or area, as a way to minimize impact and promote relaxation and calmness [10].

Research has been conducted to evaluate the effect of music on the physiological and behavioral parameters of equines. In a study by [11], new age music was played for geriatric horses for 28 days, resulting in a significant relaxation of these animals. Additionally, it was found that instrumental guitar music could positively influence Arabian racehorses when played for 5 hours daily [12]. Another study observed that the beneficial effects of music were more pronounced after 3 hours of daily exposure [13].

In the literature, most studies evaluate the impact of music over prolonged periods. Aiming to develop new application strategies, this study investigated the ability of different musical genres, previously tested and reported for equines, to promote the reduction of short-term stress responses, resulting from restraint handling in stocks (movement restriction) and social isolation.

To assess these responses, non-invasive indicators were used to express changes in physiological parameters [14]. Additionally, to provide a novel complement, facial expressions were included to analyze animals under handling stress and exposure to music. A range of facial expressions has been associated with specific emotional states, such as stress, and serves as a tool for evaluating welfare [15]. Few studies have been conducted on horses to evaluate facial expressions during potentially stressful handling situations, such as clipping [16], transportation [17], food restriction [18], and social isolation [17].

Therefore, to ensure the safe and effective use of auditory enrichment, it is necessary to conduct studies with a variety of musical genres in a range of contexts related to the typical challenges faced by horses in domestic environments. Thus, this study aims to evaluate whether auditory enrichment can attenuate short-term stress responses, resulting in a reduction of stress indicators. It also seeks to identify differences in responses between geldings and mares when exposed to different musical genres and to determine whether classical, new age and country music can promote relaxation.

## Materials and methods

### Ethics in animal research

All procedures conducted in these studies were approved by the Animal Ethics and Use Committee (CEUA) of the Federal University of Mato Grosso do Sul (UFMS, Campo Grande, Brazil) under protocol number 1.222/2022.

### Location

Experiments I and II were conducted with a one-week interval in 2023, both at the farm of the Faculty of Veterinary Medicine and Animal Science located in Terenos, MS, Central-West region of Brazil (20° 26′ 18″ S, 54° 51′ 24″ W), which has an Aw climate (tropical savanna with dry winters), according to Köppen’s classification [19].

### Microclimatic parameters

During the evaluations, meteorological variables were collected at ten-minute intervals: dry bulb temperature (DBT, °C), wet bulb temperature (WBT, °C), black globe temperature (BGT, °C), dew point temperature (DPT, °C), relative humidity (RH%), and wind speed (WS, m/s).

For the measurements, digital thermo-hygrometers (AK172®; AKSO, São Leopoldo, RS, Brazil) were used, and placed in meteorological shelters. For the black globe temperature, the same model of thermo-hygrometer was encapsulated in PVC plastic balls (0.15 cm in diameter) and painted externally with matte black paint according to the method proposed by [20]. The equipment was placed in two locations: in full sunlight and in full shade within the installation where the animals were kept during the tests. The devices were installed 1.50 meters above the ground surface, considering the variation in shadow projection and zenithal angle during the period of insolation.

Based on the collected variables, the following indices were calculated: radiant heat load (W/m^2^), according to the equation proposed by [21]; wet bulb globe temperature (WBGT, °C), according to the equation described by [22]; and thermal comfort index (TCI), as proposed by [23]. This allowed for the characterization of the environment and the thermal condition to which the animals were exposed. The mean values and standard deviations obtained in Experiments I and II were, respectively: dry bulb temperature (29 ± 1.2; 25 ± 2.8), relative humidity (70 ± 3.8; 84 ± 7.8), radiant heat load (468 ± 5.7; 422 ± 11.9), WBGT (26 ± 1.0; 22 ± 2.0) and TCI (155 ± 1.5; 162 ± 2.8).

The experimental period took place during the summer. The recorded values for air temperature, relative humidity, and radiant heat load align with the expected conditions for the region during this season, as the Aw climate is characterized by rainy summers and high temperatures [19].

The WBGT has been used to identify the risk of thermal stress in horses [24,25]. The average values obtained were < 28 °C, indicating a low risk of thermal stress [25,26]. Although the TCI exceeded 130, a value considered thermally stressful [23], the animals did not exhibit typical signs of thermal discomfort. The Pantaneiro horse, a Brazilian breed developed in the Pantanal region, has adapted over centuries to the adverse conditions of flooding and drought, as well as to the high temperatures of the region [27–29]. The environmental conditions present in the study are typical and well-tolerated within the breed’s adaptive scenario.

### Animals and housing

For Experiment I, four Pantaneiro mares were selected, with an average age of 5 ± 0.9 years and an average weight of 367.5 ± 23.6 kg. Subsequently, in Experiment II, four Pantaneiro geldings were selected, with an average age of 5 ± 1.8 years and an average weight of 388.7 ± 5.4 kg.

The animals belong to the Federal University of Mato Grosso do Sul and are maintained for research purposes. From birth, they have been kept in social groups housed in paddocks with ad libitum access to pasture (Panicum maximum cv. Tamani), mineral salt, and water, and they remained in these conditions during the experimental period when not being evaluated.

### Experimental design

The design used in both experiments was a 4x4 Latin square with experimental (musical genre) and control (no music) treatments. Four animals were individually assessed each day in the morning (7.30–10.30 a.m.). Each horse received all treatments randomly (one treatment per day) at the same time, thereby allowing each animal to serve as its own control.

The genres and corresponding musical styles chosen for auditory stimulation were evaluated in previous studies for equines, namely: Classical (Beethoven – 9th Symphony [30,31]), Country (Hank Williams Jr – Ramblin’ In My Shoes [32]), and New Age (Janet Marlow – Horsing Around [11,33]). The composer Janet Marlow, a specialist in music for animals, developed the music ‘Horsing Around’ specifically adjusted for equine hearing (My Pet Speaker, Pet Acoustics Inc).

**Fig 1:**
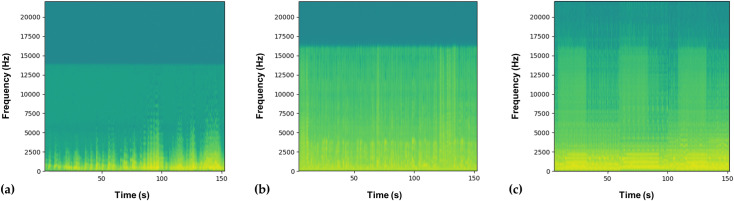
Spectrograms of the experimental treatments. **(a)** Classical (Beethoven - 9th Symphony); **(b)** Country (Hank Williams Jr - Ramblin’ In My Shoes); **(c)** New Age (Janet Marlow - Horsing Around) (created by A.C. Bini de Lima using BORIS software resources).

The spectrogram (Fig 1) is a tool for describing complex acoustic signals, allowing visualization of sound recordings in three dimensions: frequency (Hz), time (s), and amplitude (dB). Amplitude is represented by the intensity of color, with warmer or darker colors reflecting higher amplitudes [34]. This enables the observation of how the frequency spectrum of a piece of music changes over time, where higher frequencies characterize higher-pitched sounds and lower frequencies characterize lower-pitched sounds [35–37].

In the classical song ‘9th Symphony’, a maximum frequency of 15,000 Hz is noted with peaks at lower frequencies, while in the country song ‘Ramblin in my shoes’ the maximum frequency is around 16,000 Hz and continues throughout time. The new age song ‘Horsing around’ has a maximum frequency of 20,000 Hz with oscillations over time. Through the spectrograms and the acoustic signals described, it is observed that the songs of the classical and country genres present low-er-pitched sounds, and the new age genre music has higher-pitched sounds.

### Experimental protocol

Before the experimental period began, all animals underwent a habituation phase to get accustomed to the collection site, handling procedures, and evaluators. Over the course of one week, the animals were introduced to the restraining stock, where they remained for three minutes and were exposed to the equipment to be used throughout the experiment. No music was played during the habituation phase.

During the experimental period, to ensure social isolation and restriction of movement, each individual remained in a restraining stock (2.10m x 0.88m x 1.00m) located in a covered area with partially enclosed sides, preventing any contact with conspecifics. Furthermore, the area was closed off, restricting the circulation of people and mitigating acoustic environmental effects during the collection.

After the collection of each animal, the restraining stock was cleaned to ensure hygiene and prevent olfactory contact.

**Fig 2:**
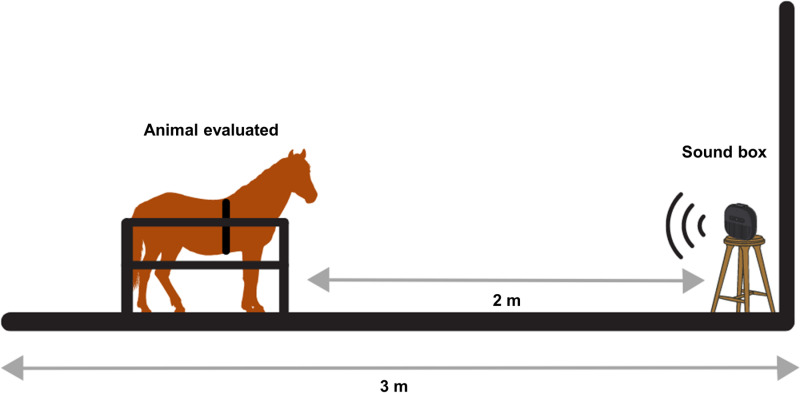
Experimental setup (created by V.C.S. da Fé using Canva resources).

For the administration of the experimental treatment, a JBL GO3 was positioned in front of the animal at a height of 0.70 m above the ground, two meters away, for 24 minutes for auditory stimulation. All music tracks were standardized in amplitude using the Decibel Meter App (Splend Apps) to ensure they reached 60–70 dB at the center of the experimental setup, a sound level corresponding to natural conditions [30,31,38] (Fig 2). During this period, the evaluator maintained a neutral facial expression to avoid potential influence on the animal’s behavior.

**Fig 3:**
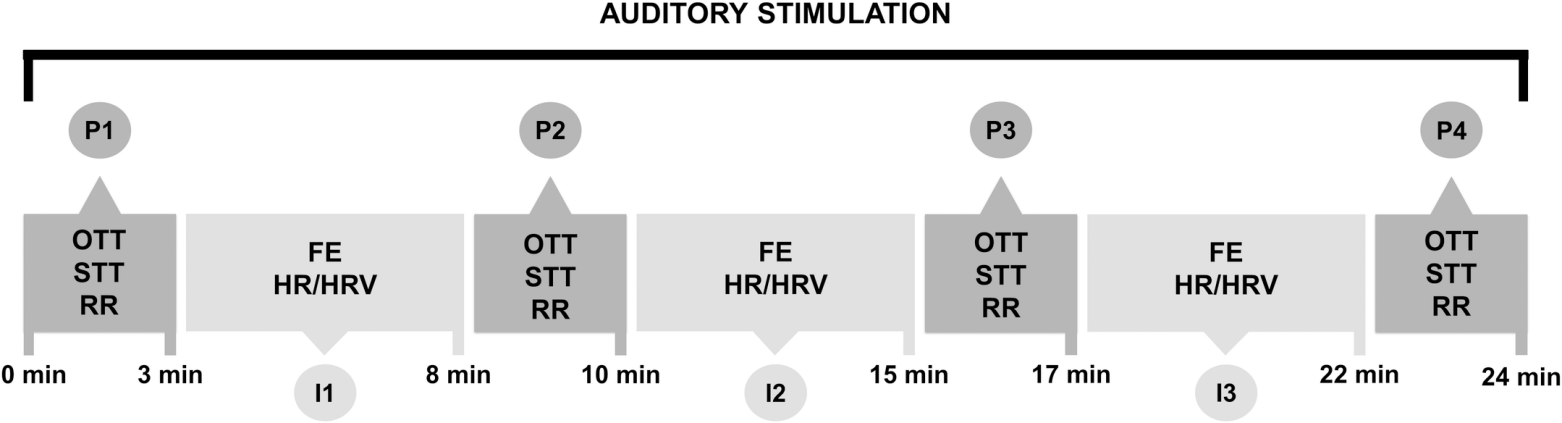
Experimental protocol. OTT = ocular temperature by infrared thermography; STT = surface temperature by infrared thermometer; RR = respiratory rate; FE = facial expressions; HR = heart rate; HRV = heart rate variability (created by V.C.S. da Fé using Canva resources).

The physiological parameters and facial expressions were assessed during the auditory stimulus presentation. The following indicators were collected at specific time points identified as P1, P2, P3, and P4: ocular temperature by infrared thermography (OTT), surface temperature by infrared thermometer (STT), and respiratory rate (RR). Facial expressions (FE) and heart rate and heart rate variability (HR/HRV) were collected continuously but assessed only at five-minute intervals identified as I1, I2, and I3 (Fig 3).

### Physiological parameters

#### Heart Rate (HR) and Heart Rate Variability (HRV).

Heart rate and heart rate variability were measured using the Polar H10 heart rate transmitter (Polar Electro Oy, Kempele, Finland). The Polar H10 was secured to an elastic belt and positioned on the thoracic region between the 4th and 5th intercostal spaces on the left side of the chest. The belt was moistened with water and adjusted on the animals 10 minutes before data collection. To enhance the transmission of electrical signals from the body to the electrodes, the hair was also cleaned with water.

Subsequently, the collected data were exported using the Elite HRV app (Elite HRV, Asheville, NC, USA), and data analysis was performed using Kubios HRV Standard software, version 3.5.0 (Kubios Oy, Kuopio, Finland). For analysis, a mean artifact correction was applied to reduce error across the entire sample set, following the methodology of [39], which allows for a correction of 15% or less. Out of the 32 samples collected, only 2 had values greater than 5%, specifically 5.29% and 8.06%. Three five-minute intervals were then selected for the analysis of HRV variables and mean HR. The following frequency domain HRV variables were selected: low-frequency power (LF; nu), high-frequency power (HF; nu), and low-frequency to high-frequency ratio (LF/HF).

#### Respiratory Rate (RR).

The respiratory rate, measured in movements per minute (mpm), was determined by counting flank movements over a period of 30 seconds. The value obtained was then multiplied by 2 to calculate the respiratory rate per minute.

#### Ocular Temperature by Infrared Thermography (OTT).

Thermographic images of the eye were collected to measure the superficial temperature in the medial canthus region, as proposed by [14]. Images were captured from the left side of the animal at a 90° angle to the sagittal plane, directed towards the eye at a distance of 0.5 meters, using an infrared thermographic camera (S60, Caterpillar FLIR, Vernon Hills, IL, USA). The camera’s emissivity was set to 0.98, a value corresponding to that used for tissue surfaces. The FLIR Tools software, version 6.4.18039.1003 (FLIR Systems Inc, Oregon, USA), was used to analyze the images.

#### Surface Temperature by Infrared Thermometer (STT).

**Fig 4:**
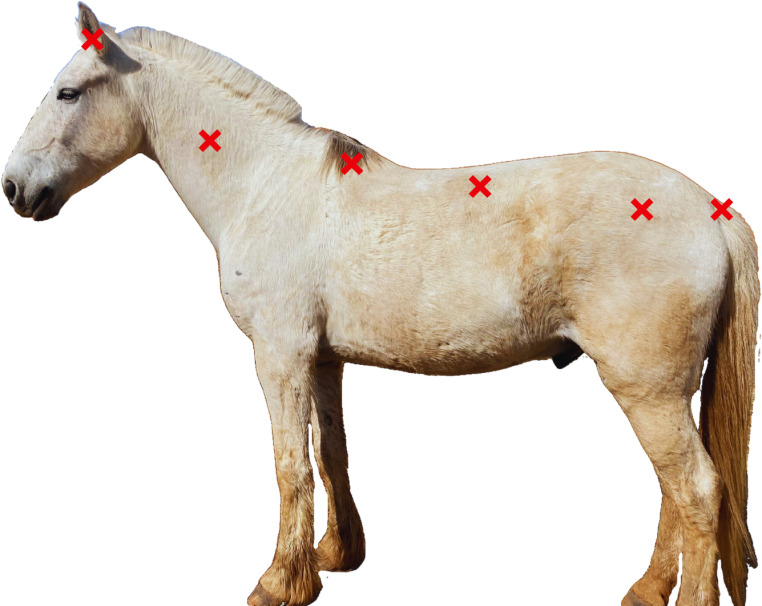
Illustration of the temperature collection points: auricular, and superficial points of the neck, withers, back, croup, and tail insertion (photo credit and creation by M.S.P. Hernandes).

The surface temperature was measured using a portable digital infrared thermometer (Mult Temp Portable, Incoterm, Porto Alegre, RS, Brazil). Measurements were taken at six points on the left side of the animals (Fig 4), as follows:

- Auricular: Central point of the ear cavity on the left ear of the animal [40];
- Neck Surface: Midpoint of the line connecting the most caudal point of the Atlas bone with the most prominent point of the scapular crest. This region of the neck has a rich blood supply, and this methodology was based on the studies by [41];
- Withers Surface: Located on the midline, an odd region situated cranially on the upper dorsal part of the trunk [42];
- Back Surface: Midpoint of the line connecting the most caudal point of the scapular cartilage with the iliac tuberosity. This point shows considerable thermal variability and is considered representative of the average body surface, according to the methodology adopted by [43];
- Croup Surface: Midpoint of the line connecting the iliac tuberosity with the tail insertion, following the methodology proposed by [43];
- Tail Insertion Surface: Unique point at the junction of the sacral and caudal vertebrae, projection of the coccygeal artery 10 cm from the start of the tail on the ventral portion [43].

The infrared thermometer was aimed at the animal’s coat from a distance of 1 meter, except for the auricular and tail insertion measurements, which were collected from a distance of 0.5 meters. For assessment, auricular temperature and body surface temperature (obtained from the average of all collected points excluding the auricular point) were selected.

### Facial Expressions (FE)

During the period the animal was restrained in the stock, video recordings were made using a Canon EOS SL3 digital camera (Canon Inc., São Paulo, SP, Brazil). The resolution was set to 1080p at 30 fps, and the videos were exported in mp4 format. The camera was positioned 1.5 meters away at a 45° angle relative to the medial plane of the horse.

EquiFACS, as described by [15], was used for facial expression assessment. For this study, four Action Units (AUs), seven Action Descriptors (ADs), and one Visibility Code (VC) were selected, as outlined in Table 1. These codes correspond to facial expressions that have been shown to be relevant for assessing horses under stress conditions in previous studies [16–18].

**Table 1:** Summary of EquiFACS codes selected for the assessment of facial expressions in horses subjected to social isolation and movement restriction.

I. Action Unit (AU)	
**Codes**	**Minimum criteria to code**
*Eye Closure* (AU143)	Both eyelids must move together to cover the eye, and this action lasts for more than half a second.
*Blink* (AU145)	Both eyelids must move together to cover the eye, and this action should be reversed within half a second.
*Half Blink* (AU47)	Reduction in eye opening
*Inner Brow Raiser* (AU101)	Dorsal movement of the skin above the inner region of the eye
**II. Action Descriptors (AD)**	
*Eye White Increase* (AD1)	An increase in the percentage of visible sclera
*Tongue Show* (AD19)	The tongue is exposed and extends beyond the teeth
*Nostril Dilator* (AD38)	An increase in nostril aperture
*Chewing* (AD81)	Minimum criteria for coding not described
*Ears Forward* (EAD101)	Rostral rotation of the auricular pavilion
*Ear Rotator* (EAD104)	The auricular pavilion rotates caudally
*Full face not visible* (VC73)	The entire face is out of view or cannot be clearly seen

The designated AUs represent the contraction of a specific facial muscle (or muscle group) and the resulting facial movements [44]. The ADs are also used for facial movements, but these are general movements where the underlying muscle cannot be identified, or they result from a different muscle group [15].

All videos were reviewed by a single EquiFACS-certified coder using BORIS software, version 6.0.6 (Friard and Gamba, University of Turin, Turin, Italy), with inter-observer agreement >70% compared to experienced coders and 95% intra-observer agreement. Intraclass correlation coefficients (ICCs) ranged from 0.99 to 1.00, demonstrating high concordance. Coding was performed on 5-minute clips obtained during the animals’ time in the stock (I1, I2, and I3).

Continuous focal sampling was used to record the facial expressions of each horse. The videos were initially observed at normal speed and then reviewed at least three more times in slow motion or frame-by-frame. The frequency per minute of each of the proposed codes was assessed, except for VC73, which was measured in seconds. VC73 was used to indicate how long the entire face was not visible for coding.

This approach allowed for monitoring the duration for which the face was in a visible position for coding, or not, during each 5-minute interval. To calculate the frequency per minute of AUs and ADs, only the time in which the face was in a visible position for coding was considered:


FrequencyperminuteofAUorAD=Totalnumberofrecords/(5minutes−VC73)

### Statistical description

All statistical analyses were performed using R software with the RStudio integrated development environment (Version 4.1.0 (2021-06-29), RStudio, Inc.). The functions and packages utilized are presented in the format ‘package::function’ corresponding to the R programming language. A significance level of 5% was considered for all tests.

Initially, Pearson’s correlation coefficient (Hmisc::rcorr) was estimated among variables derived from heart rate (mean heart rate, LF, HF, and LF/HF). Of those highly correlated, only one (LF/HF) was selected for use in the inferential stage (modeling), based on its lower variance and greater biological relevance to the study’s aim.

Subsequently, an inferential analysis was conducted. To identify differences between treatments (classical vs country vs control vs new age) and over time (P1 vs P2 vs P3 vs P4 or I1 vs I2 vs I3), multilevel linear models (lme4::lmer) were fitted for response variables (LF/HF, respiratory rate, auricular temperature, ocular temperature, body temperature, *inner brow raiser* - AU101, *nostril dilator* - AD38, *ears forward* - EAD101, *ear rotator* - EAD104, *chewing* - AD81, *blink* - AU145, and *half blink* - AU47) where model residuals adhered to normality as assessed by the Cramer-Von Mises test (nortest::cvm.test). Multiple comparisons in the post-hoc test were conducted using Tukey’s procedure (lsmeans::lsmeans and multcomp::cld).

The interaction of collection points or intervals with treatments and the order were used as fixed effects in the modeling, while horses were used as random effects to control individual variation.

Mean heart rate, *eye white increase* (AD1), *tongue show* (AD19), and *eye closure* (AU143) did not meet the normality assumption even after transformations, preventing their modeling. Therefore, comparisons between each treatment at the same interval and comparisons over time for each treatment were conducted using the Friedman test (stats::friedman.test and PMCMR-plus::frdAllPairsNemenyiTest). Results were illustrated with box plots (ggplot2::ggplot and ggplot2::geom_boxplot).

Subsequently, an exploratory analysis was conducted. To analyze the multivariate dynamics between facial expressions, principal component analysis (PCA) was performed based on a correlation matrix (stats::princomp). The optimal number of principal components (PCs) to retain in PCA was determined by Horn’s parallel analysis (‘psych::fa.parallel’). To establish associations between each variable and the PCs, loadings greater than 0.40 or less than -0.40 were used as the criterion. The results were illustrated with two biplots, where observations were color-coded according to treatments for qualitative (visual) assessment of their distribution.

To ensure reliability in the coding of facial expressions, the intraclass correlation coefficient (ICC) was calculated (irr::icc()) using data collected from ten randomly selected 5-minute videos of the study animals. The experimental design was set as “two-way” and “agreement,” resulting in the concordance between multiple measurements taken by the same observer on different occasions.

## Results

### Experiment I

The mean heart rate of the mares decreased (p < 0.05) across intervals (I1, I2, I3) for both classical and country music treatments (Table 2). The LF/HF ratio did not differ (p > 0.05) between treatments or intervals.

**Table 2:** Mean and standard deviation, or median and interquartile range, of the average heart rate (bpm) and LF/HF ratio for the control, classical, new age, and country treatments in mares.

Parameters	Treatments	Intervals		
		I1	I2	I3
Heart Rate (bpm)	Control	49.72(6.61)	48.39(6.57)	51.08(6.27)
	Classical	**48.66(3.33)** ^**a**^	**48.29(2.66)** ^**ab**^	**47.25(2.54)** ^**b**^
	New Age	46.00(2.65)	48.77(2.18)	46.35(2.00)
	Country	**45.86(3.87)** ^**a**^	**45.52(3.64)** ^**ab**^	**44.08(2.67)** ^**b**^
LF/HF	Control	2.98 ± 0.97	2.49 ± 0.85	3.09 ± 1.41
	Classical	2.27 ± 0.90	2.62 ± 0.59	2.57 ± 1.30
	New Age	2.31 ± 0.46	1.99 ± 1.21	2.59 ± 1.11
	Country	2.65 ± 1.12	2.69 ± 0.77	2.67 ± 0.85

Different lowercase letters indicate a statistically significant difference according to the post-hoc test across intervals for the same treatment (p < 0.05), where a > b; Different uppercase letters indicate a statistically significant difference according to the post-hoc test between treatments at the same interval (p < 0.05), where A > B.

Body and auricular temperatures differed (p < 0.05) between time points (P1 and P4) in the classical treatment (Table 3). Respiratory rate and ocular temperature did not differ (p > 0.05) between treatments or time points.

**Table 3:** Physiological parameters of the control, classical, new age, and country treatments in mares across four-time points.

Parameters	Treatments	Time Points			
		P1	P2	P3	P4
Body Temperature	Control	32.3 ± 0.52	32.5 ± 0.56	32.8 ± 0.55	32.6 ± 1.10
	Classical	**32.3 ± 1.08** ^**b**^	**32.6 ± 0.96** ^**ab**^	**32.7 ± 0.80** ^**ab**^	**33.2 ± 1.13** ^**a**^
	New Age	32.4 ± 0.55	32.6 ± 0.74	32.6 ± 0.89	32.9 ± 1.12
	Country	32.2 ± 0.65	32.6 ± 0.40	32.6 ± 0.46	32.9 ± 0.47
Auricular Temperature	Control	31.9 ± 0.68	31.8 ± 0.69	32.0 ± 1.03	32.0 ± 1.04
	Classical	**31.7 ± 0.74** ^**b**^	**32.4 ± 0.90** ^**ab**^	**32.5 ± 0.88** ^**ab**^	**32.9 ± 1.09** ^**a**^
	New Age	31.6 ± 0.73	31.8 ± 0.64	31.8 ± 1.13	32.2 ± 1.38
	Country	31.4 ± 0.43	31.5 ± 1.02	31.8 ± 0.76	32.1 ± 0.77
Respiratory Rate	Control	25.0 ± 5.03	25.0 ± 8.25	23.0 ± 8.25	24.0 ± 7.30
	Classical	24.0 ± 5.66	21.0 ± 5.03	23.0 ± 6.00	19.0 ± 5.03
	New Age	24.0 ± 4.62	26.0 ± 5.66	24.0 ± 5.66	22.0 ± 2.31
	Country	22.0 ± 5.03	22.0 ± 5.16	28.0 ± 10.80	25.0 ± 5.03
Ocular Temperature	Control	30.4 ± 0.55	29.5 ± 0.89	29.4 ± 0.86	29.9 ± 0.84
	Classical	30.2 ± 0.77	29.8 ± 0.55	29.5 ± 1.01	30.4 ± 1.35
	New Age	30.1 ± 1.05	30.0 ± 0.66	29.9 ± 0.39	29.9 ± 1.33
	Country	29.7 ± 0.82	29.8 ± 0.39	30.2 ± 1.32	29.6 ± 0.40

Different lowercase letters indicate a statistically significant difference according to the post-hoc test across time points for the same treatment (p < 0.05), where a > b > c > d. Different uppercase letters indicate significant statistical differences from the post-hoc test between treatments at the same time point (p < 0.05), where A > B.

**Fig 5:**
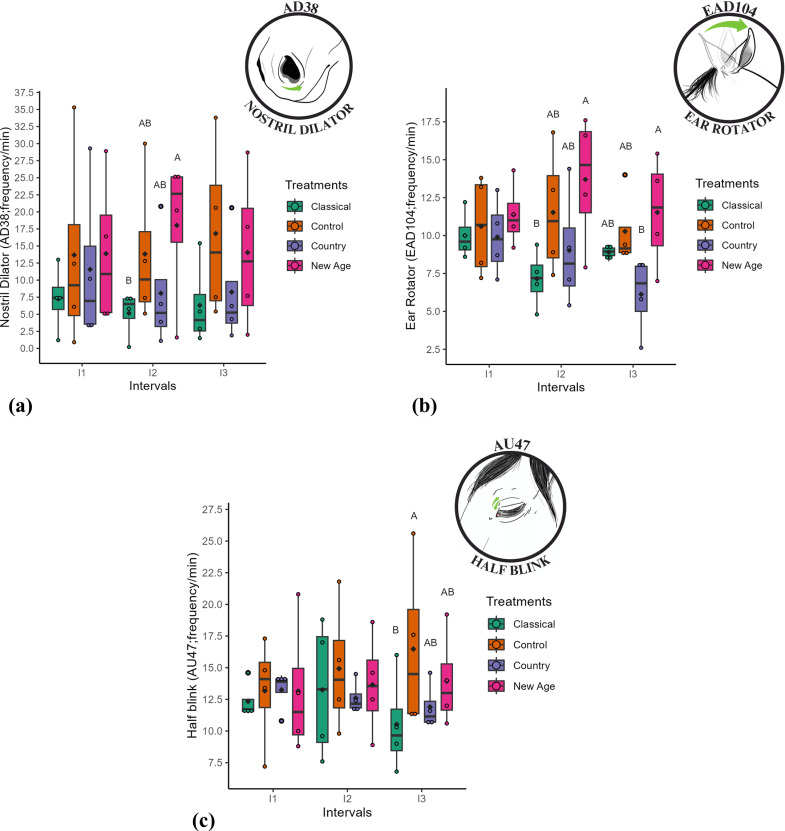
Box plots: (a) *Nostril dilator* (AD38; frequency/min); (b) *Ear rotator* (EAD104; frequency/min); (c) *Half blink* (AU47; frequency/min) in mares under the classical, control, country, and new age treatments across intervals. Different uppercase letters indicate significant statistical differences from post-hoc tests between treatments within the same interval (p < 0.05), where A > B; the black diamond represents the mean; each colored circle, corresponding to the respective treatment colors, represents an individual animal.

The frequency of facial action descriptors *nostril dilation* (AD38) and *ear rotator* (EAD104) in the second interval (I2) was higher (p < 0.05) for the new age treatment and lower for the classical treatment (Table 4; Fig 5). In the third interval (I3), the frequency of the *ear rotator* (EAD104) expression was higher (p < 0.05) for the new age treatment and lower for the country treatment.

**Table 4:** Mean and standard deviation, or median and interquartile range of the frequency per minute of facial parameters for the control, classical, new age, and country treatments in mares across three intervals.

Parameters	Treatments	Intervals		
		I1	I2	I3
*Nostril Dilator* (AD38)	Control	13.70 ± 15.20	**13.80 ± 11.30** ^**AB**^	16.80 ± 13.20
	Classical	7.25 ± 4.82	**5.15 ± 3.38** ^**B**^	6.30 ± 6.28
	New Age	13.90 ± 11.30	**18.00 ± 11.20** ^**A**^	14.00 ± 11.80
	Country	11.60 ± 12.20	**8.07 ± 8.77** ^**AB**^	8.25 ± 8.42
*Ear Rotator* (EAD104)	Control	10.60 ± 3.38	**11.50 ± 4.24** ^**AB**^	**10.30 ± 2.50** ^**AB**^
	Classical	10.00 ± 1.57	**7.15 ± 1.91** ^**B**^	**8.90 ± 0.47** ^**AB**^
	New Age	11.40 ± 2.15	**13.70 ± 4.41** ^**A**^	**11.50 ± 3.73** ^**A**^
	Country	9.90 ± 2.56	**9.02 ± 3.91** ^**AB**^	**6.12 ± 2.58** ^**B**^
*Half Blink* (AU47)	Control	13.20 ± 4.30	14.90 ± 5.16	**16.50 ± 6.76** ^**A**^
	Classical	12.40 ± 1.51	13.20 ± 5.48	**10.50 ± 3.93** ^**B**^
	New Age	13.20 ± 5.40	13.60 ± 4.05	**14.00 ± 3.77** ^**AB**^
	Country	13.20 ± 1.65	12.60 ± 1.31	**11.90 ± 1.85** ^**AB**^
*Inner Brow Raiser* (AU101)	Control	2.20 ± 1.09	1.80 ± 2.16	2.12 ± 1.79
	Classical	1.40 ± 1.18	1.35 ± 1.11	1.23 ± 0.97
	New Age	2.58 ± 2.55	3.10 ± 3.15	2.70 ± 2.25
	Country	1.30 ± 0.74	1.08 ± 0.854	1.10 ± 0.62
*Ears Forward* (EAD101)	Control	10.40 ± 3.42	12.70 ± 5.60	8.25 ± 1.32
	Classical	9.80 ± 4.87	6.90 ± 3.97	7.30 ± 1.42
	New Age	11.40 ± 2.28	12.10 ± 3.37	8.85 ± 2.52
	Country	9.40 ± 5.35	7.25 ± 1.22	5.32 ± 3.23
*Blink* (AU145)	Control	5.62 ± 2.93	3.88 ± 1.91	5.85 ± 1.42
	Classical	5.68 ± 2.39	5.85 ± 2.59	5.98 ± 2.52
	New Age	3.85 ± 1.88	5.38 ± 1.88	6.28 ± 2.62
	Country	6.92 ± 3.51	4.50 ± 2.53	5.28 ± 2.21
*Chewing* (AD81)	Control	1.23 ± 0.65	0.72 ± 0.51	0.50 ± 0.48
	Classical	0.40 ± 0.28	0.50 ± 0.11	0.15 ± 0.10
	New Age	0.65 ± 0.66	1.12 ± 0.15	0.90 ± 0.42
	Country	0.50 ± 0.26	0.97 ± 0.98	0.97 ± 1.16
*Eye White* *Increase* (AD1)	Control	0.30(0.40)	0.30(0.65)	0.10(0.30)
	Classical	0.00(0.10)	0.20(0.10)	0.10(0.20)
	New Age	0.30(0.65)	0.00(0.52)	0.00(0.05)
	Country	0.40(0.15)	0.20(0.40)	0.20(0.55)
*Tongue Show* (AD19)	Control	0.00(0.25)	0.00 (0.05)	0.10(0.30)
	Classical	0.00(2.40)	0.10(1.40)	0.00(0.20)
	New Age	0.30(0.60)	0.00(1.62)	0.10(0.60)
	Country	0.10(2.28)	0.00(1.12)	0.10(0.77)
*Eye Closure* (AU143)	Control	0.20(0.20)	0.10(0.42)	0.20(0.50)
	Classical	0.00(0.00)	0.00(0.60)	0.00(1.5)
	New Age	0.10(0.30)	0.00(0.00)	0.00(0.00)
	Country	0.00(0.00)	0.00(0.10)	0.20(0.15)

Different lowercase letters indicate a statistically significant difference according to the post-hoc test across intervals for the same treatment (p < 0.05), where a > b; Different uppercase letters indicate a statistically significant difference according to the post-hoc test between treatments at the same interval (p < 0.05), where A > B.

The frequency of the facial action unit *half blink* (AU47) during interval three (I3) was higher (p < 0.05) for the control treatment, and lower for the classical treatment. The new age and country treatments did not differ from each other.

Horn’s parallel analysis indicated the retention of the first and second principal components (PCs) out of a total of 5 PCs generated by the principal component analysis (PCA), thus only these were analyzed. The PCA loadings represent the level of association between a variable and a particular PC, with loadings further from zero indicating a higher level of positive (1.00) or negative (-1.00) association. Therefore, a set of variables positively associated with a PC exhibits similar dynamics, meaning they all increase or decrease concurrently.

Associations were determined using a cut-off loading value of 0.40, either positive or negative. PC1 alone accounted for the largest portion (31.4%) of the total variance in the data. The variables *ears forward* (EAD101), *ear rotator* (EAD104), *half blink* (AU47), *eye white increase* (AD1), *nostril dilator* (AD38), and *chewing* (AD81) showed positive associations (loading values > 0.40) with PC1 (Table 5). Thus, the increase or decrease in these facial expressions occurred concurrently across treatments. *Nostril dilator* (AD38) exhibited the loading value furthest from zero on PC1, indicating it as the facial expression with the greatest variation (importance) on PC1. *Blink* (AU145), *tongue show* (AD19), and *eye closure* (AU143) showed a positive association (loading values > 0.40) with PC2. In contrast, the *inner brow raiser* (AU101) was the only facial expression that exhibited a negative association with PC2.

**Table 5:** Loading values, eigenvalues, and variance from the principal component analysis of mares (PC = principal component; values in bold indicate loadings greater than 0.40 or less than -0.40, indicating the variable’s association with the PC).

Variables	PC1	PC2
*Ears Forward* (EAD101)	**0.741**	0.152
*Ear Rotator* (EAD104)	**0.812**	-0.058
*Inner Brow Raiser* (AU101)	0.369	**-0.535**
*Half Blink* (AU47)	**0.596**	0.314
*Blink* (AU145)	-0.045	**0.612**
*Eye Closure* (AU143)	-0.031	**0.462**
*Eye White Increase* (AD1)	**0.504**	-0.400
*Nostril Dilator* (AD38)	**0.875**	-0.026
*Chewing* (AD81)	**0.623**	0.147
*Tongue Show* (AD19)	0.174	**0.702**
Eigenvalues	3.144	1.678
Variance	31.441	16.784
Cumulative variance	31.441	48.225

**Fig 6:**
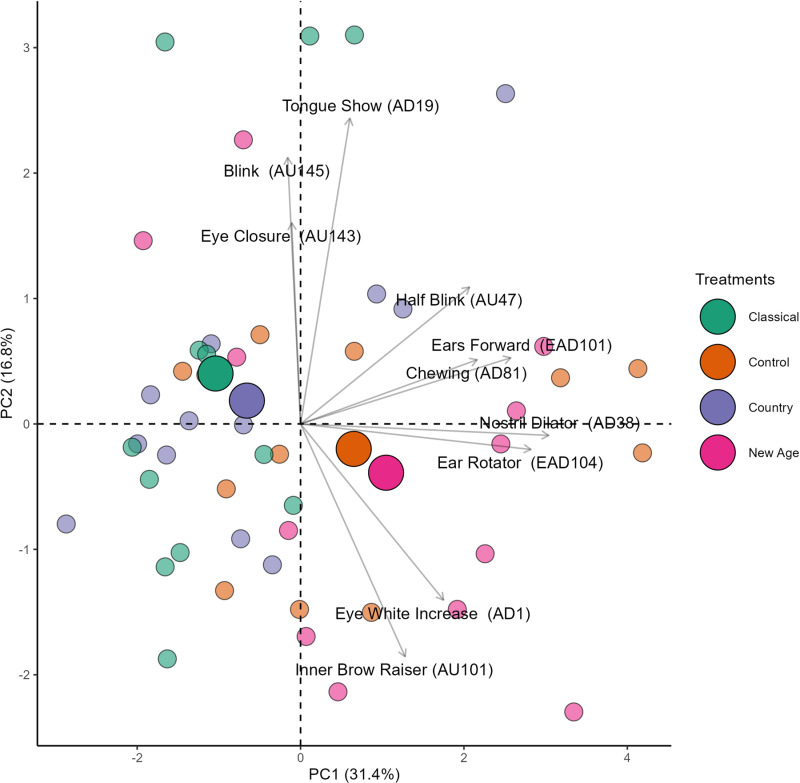
Two-dimensional biplot of the principal component analysis (PCA) of mares, displaying facial expressions and observations categorized by treatment (smaller circles represent individual observations; while larger circles denote the centroid for each treatment; the centroid represents the center of mass calculated from the polygon formed by interpolating the smaller circles of the same color; arrows indicate the vectors for each facial expression).

Fig 6 separates the evaluations of mares across classical, control, country, and new age treatments, represented by different colors. One of the features of this figure is the centroid (larger circle), which indicates the center of mass derived from the polygon (geometric shape) formed by interpolating the evaluations (smaller circles) of the same color. Visual inspection suggests that the centroids for the classical and country treatments are distinct from those for the new age and control treatments, indicating that facial expressions were able to differentiate between the treatments.

Moreover, the centroids for the new age and control treatments are located in the lower right quadrant along with the vectors for *ear rotator* (EAD104), *inner brow raiser* (AU101), *eye white increase* (AD1), and *nostril dilator* (AD38). In contrast, the centroids for the classical and country treatments are positioned in the upper left quadrant along with the vectors for *eye closure* (AU143) and *blink* (AU145), suggesting a higher occurrence of these facial expressions in the respective treatments. These results explore a possible multifaceted dynamic of facial expressions that were able to distinguish treatments in the previous inferential analyses (modeling).

### Experiment II

The average heart rate (bpm) and LF/HF ratio in geldings did not differ (p > 0.05) between treatments or across intervals (Table 6).

**Table 6:** Mean and standard deviation, or median and interquartile range, of the average heart rate (bpm) and LF/HF ratio for the control, classical, new age, and country treatments in geldings.

Parameters	Treatments	Intervals		
		I1	I2	I3
Heart Rate (bpm)	Control	38.05(8.84)	37.87(7.56)	37.13(5.77)
	Classical	38.81(3.21)	40.42(6.17)	42.04(7.06)
	New Age	41.07(4.24)	36.58(11.40)	43.15(4.67)
	Country	39.93(1.78)	39.64(2.70)	41.03(3.21)
LF/HF	Control	1.14 ± 1.56	1.66 ± 0.43	2.30 ± 0.59
	Classical	1.06 ± 0.95	1.23 ± 1.01	1.57 ± 9.59
	New Age	1.22 ± 0.93	1.15 ± 0.74	2.98 ± 6.07
	Country	1.56 ± 1.27	1.17 ± 0.74	2.74 ± 1.06

Different lowercase letters indicate a statistically significant difference according to the post-hoc test across intervals for the same treatment (p < 0.05), where a > b; Different uppercase letters indicate a statistically significant difference according to the post-hoc test between treatments at the same interval (p < 0.05), where A > B.

The respiratory rate at the first time point (P1) increased (p < 0.05), differing between the control and new age treatments. Ocular temperature increased (p < 0.05) between time points (P1 and P3) in the control and country treatments (Table 7).

**Table 7:** Physiological parameters of the control, classical, new age, and country treatments in geldings across four-time points.

Parameters	Treatments	Time Points			
		P1	P2	P3	P1
Respiratory Rate	Control	**16.0 ± 3.27** ^**B**^	19.0 ± 2.00	20.0 ± 4.62	21.0 ± 3.83
	Classical	**22.0 ± 4.00** ^**AB**^	17.0 ± 2.00	17.0 ± 3.83	18.0 ± 2.31
	New Age	**25.0 ± 3.83** ^**A**^	22.0 ± 7.66	19.0 ± 8.87	20.0 ± 9.80
	Country	**19.0 ± 6.83** ^**AB**^	22.0 ± 5.16	21.0 ± 5.03	19.0 ± 3.83
Ocular Temperature	Control	**28.9 ± 1.67** ^**b**^	**29.8 ± 1.02** ^**ab**^	**30.7 ± 0.88** ^**a**^	**29.9 ± 0.41** ^**ab**^
	Classical	29.4 ± 0.73	30.1 ± 1.21	29.8 ± 0.85	30.2 ± 0.66
	New Age	29.4 ± 0.70	30.8 ± 0.68	29.8 ± 0.90	29.7 ± 2.07
	Country	**28.5 ± 1.27** ^**b**^	**29.8 ± 0.72** ^**ab**^	**30.3 ± 0.28** ^**a**^	**30.2 ± 1.10** ^**ab**^
Auricular Temperature	Control	30.3 ± 2.49	30.8 ± 2.20	30.4 ± 1.85	30.7 ± 2.69
	Classical	30.5 ± 1.52	31.5 ± 1.02	30.8 ± 2.00	31.0 ± 1.09
	New Age	30.4 ± 3.69	30.8 ± 2.38	30.8 ± 1.73	31.4 ± 1.43
	Country	31.0 ± 2.93	31.0 ± 2.13	31.2 ± 2.14	31.2 ± 1.84
Body Temperature	Control	31.7 ± 1.85	32.0 ± 1.24	31.9 ± 1.36	32.1 ± 1.90
	Classical	31.5 ± 1.33	31.9 ± 1.14	31.6 ± 1.08	31.8 ± 1.06
	New Age	31.2 ± 2.08	31.7 ± 1.77	31.4 ± 1.68	31.9 ± 1.65
	Country	32.0 ± 2.47	31.7 ± 1.89	31.8 ± 1.92	31.9 ± 1.99

Different lowercase letters indicate a statistically significant difference according to the post-hoc test across time points for the same treatment (p < 0.05), where a > b > c > d; Different uppercase letters indicate significant statistical differences from the post-hoc test between treatments at the same time point (p < 0.05), where A > B.

**Fig 7:**
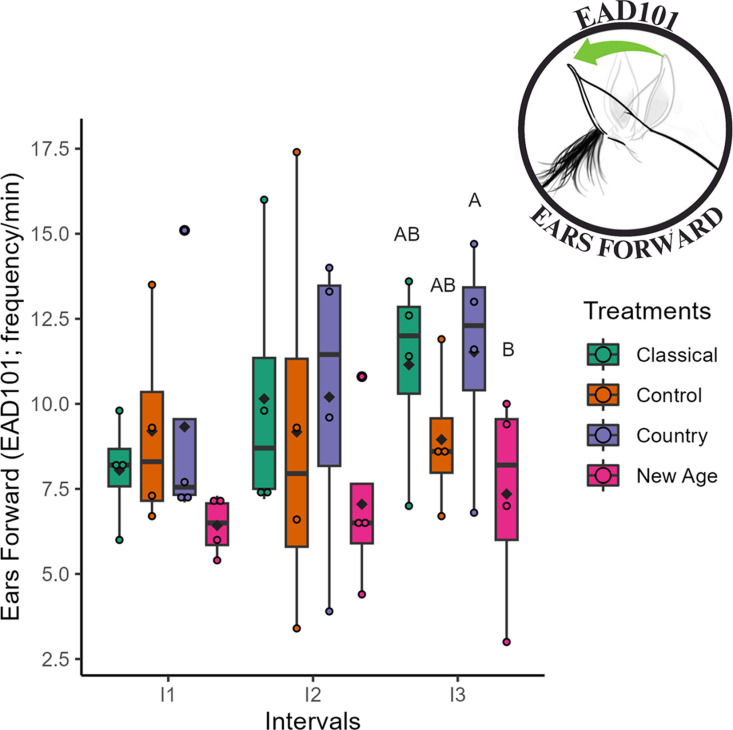
Box plot of the facial action descriptor *ears forward* (EAD101) in geldings for the classical, control, country, and new age treatments across intervals. Different uppercase letters indicate statistically significant differences from the post-hoc test between treatments within the same interval (p < 0.05), with A > B; the black diamond represents the mean; each colored circle corresponds to an animal, with colors representing the respective treatments.

The frequency of the action descriptor *ears forward* (EAD101) in interval three (I3) was higher (p < 0.05) for the country treatment and lower for the new age treatment (Table 8; Fig 7). The action unit *eye closure* (AU143) was not selected due to its low frequency.

**Table 8:** Mean and standard deviation, or median and interquartile range of the frequency per minute of facial parameters for the control, classical, new age, and country treatments in geldings across three intervals.

Parameters	Treatments	Intervals		
		I1	I2	I3
*Ears Forward* (EAD101)	Control	9.20 ± 3.07	9.17 ± 5.99	**8.95 ± 2.17** ^**AB**^
	Classical	8.05 ± 1.56	10.20 ± 4.06	**11.20 ± 2.91** ^**AB**^
	New Age	6.42 ± 0.88	7.05 ± 2.69	**7.35 ± 3.18** ^**B**^
	Country	9.32 ± 3.86	10.20 ± 4.62	**11.50 ± 3.40** ^**A**^
*Nostril Dilator* (AD38)	Control	6.55 ± 2.79	7.18 ± 2.56	11.80 ± 7.20
	Classical	9.00 ± 8.38	7.92 ± 6.43	7.95 ± 6.28
	New Age	8.12 ± 8.75	7.85 ± 8.87	9.70 ± 7.26
	Country	9.05 ± 5.64	7.15 ± 5.08	8.60 ± 3.52
*Inner Brow Raiser* (AU101)	Control	1.90 ± 0.92	1.88 ± 1.23	2.30 ± 1.91
	Classical	1.27 ± 0.61	2.17 ± 0.75	2.68 ± 1.97
	New Age	1.98 ± 1.03	1.98 ± 0.92	2.05 ± 1.68
	Country	1.65 ± 1.23	0.80 ± 0.59	2.10 ± 1.41
*Ear Rotator* (EAD104)	Control	10.20 ± 4.07	10.90 ± 5.31	9.85 ± 1.94
	Classical	10.70 ± 1.11	11.20 ± 1.38	11.80 ± 1.24
	New Age	7.82 ± 2.38	8.35 ± 1.98	8.10 ± 0.66
	Country	10.7 ± 4.92	11.80 ± 7.92	10.80 ± 5.06
*Chewing* (AD81)	Control	0.77 ± 0.79	1.10 ± 0.70	0.87 ± 0.90
	Classical	0.90 ± 0.62	0.75 ± 0.34	0.35 ± 0.19
	New Age	1.12 ± 0.73	0.75 ± 0.47	0.55 ± 0.19
	Country	0.85 ± 0.66	1.12 ± 0.73	0.60 ± 0.37
*Blink* (AU145)	Control	5.65 ± 1.84	6.98 ± 3.13	6.82 ± 1.50
	Classical	7.10 ± 3.11	6.32 ± 2.85	6.95 ± 3.81
	New Age	5.78 ± 0.56	6.70 ± 2.37	6.90 ± 3.40
	Country	7.20 ± 4.56	6.15 ± 4.29	5.60 ± 2.47
*Half Blink* (AU47)	Control	14.90 ± 3.38	13.50 ± 4.57	13.40 ± 2.07
	Classical	11.80 ± 4.05	13.40 ± 6.38	11.30 ± 2.90
	New Age	14.60 ± 3.33	15.60 ± 2.29	14.30 ± 5.02
	Country	13.20 ± 3.76	13.40 ± 3.36	15.00 ± 2.06
*Eye White Increase* (AD1)	Control	0.40(0.20)	0.20(0.15)	0.30(0.45)
	Classical	0.50(0.80)	0.00(0.57)	0.00(0.15)
	New Age	0.50(0.45)	0.30(0.25)	0.20(0.55)
	Country	0.30(0.30)	0.30(0.65)	0.20(0.10)
*Tongue Show* (AD19)	Control	0.75(1.27)	0.20 (0.27)	0.20(0.47)
	Classical	0.30(1.70)	0.00(0.20)	0.10(0.25)
	New Age	0.80(2.25)	0.60(1.05)	0.50(1.15)
	Country	0.40(1.30)	0.30(0.25)	0.20(0.40)

Different lowercase letters indicate a statistically significant difference according to the post-hoc test across intervals for the same treatment (p < 0.05), where a > b; Different uppercase letters indicate a statistically significant difference according to the post-hoc test between treatments at the same interval (p < 0.05), where A > B.

Horn’s parallel analysis indicated the retention of the first and second principal components (PCs) out of a total of 5 PCs generated by principal component analysis (PCA); thus, only these two components were analyzed.

The PC1 alone accounted for the largest portion (28.8%) of the total data variation, with *ears forward* (EAD101), *ear rotator* (EAD104), *inner brow raiser* (AU101), *half blink* (AU47), and *chewing* (AD81) showing a positive association (loading values >0.40) with PC1 (Table 9).

**Table 9:** Loadings, eigenvalues, and variance from the principal component analysis of geldings (PC = principal component; values greater than 0.40 or less than -0.40 are in bold, indicating a strong association of the variable with the PC).

Variable	PC1	PC2
*Ears Forward* (EAD101)	**0.785**	-0.287
*Ear Rotator* (EAD104)	**0.689**	-0.120
*Inner Brow Raiser* (AU101)	**0.686**	-0.110
*Half Blink* (AU47)	**0.443**	-0.059
*Blink* (AU145)	-0.302	**0.573**
*Eye White Increase* (AD1)	0.376	-0.390
*Nostril Dilator* (AD38)	-0.030	-0.386
*Chewing* (AD81)	**0.678**	0.386
*Tongue Show* (AD19)	0.377	**0.746**
Eigenvalues	2.595	1.808
Variance	28.834	20.087
Cumulative variance	28.834	48.921

Consequently, the decrease or increase in these facial expressions occurred concurrently across treatments. *Ears forward* (EAD101) exhibited the most extreme loading value on PC1, which can be interpreted as the facial expression with the greatest variation (importance) on PC1. *Blink* (AU145) and *tongue show* (AD19) showed a positive association (loading values >0.40) with PC2.

**Fig 8:**
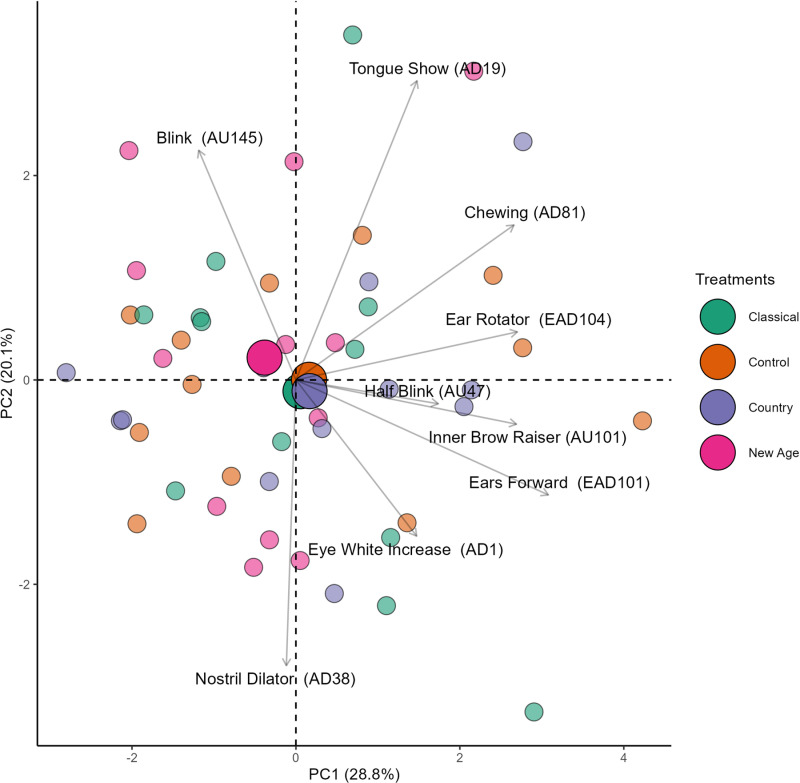
Two-dimensional biplot of the principal component analysis (PCA) of geldings, displaying facial expressions and observations categorized by treatment (smaller circles represent individual observations; while larger circles denote the centroid for each treatment; the centroid represents the center of mass calculated from the polygon formed by interpolating the smaller circles of the same color; arrows indicate the vectors for each facial expression).

Fig 8 separates the evaluations of horses across the classical, control, country, and new age treatments using different colors. A visual inspection reveals that the centroids of the classical, country, and control treatments are distinct from the centroids of the new age treatment, indicating that facial expressions were able to differentiate the treatments.

Moreover, the centroids of the classical, country, and control treatments are located in the lower-right quadrant, overlapping and close to the center of the quadrants, along with the vectors for *inner brow raiser* (AU101), *eye white increase* (AD1), *ears forward* (EAD101), and *half blink* (AU47). In contrast, the centroid of the new age treatment is positioned in the upper-left quadrant, along with the vector for *blink* (AU145), suggesting a higher occurrence of these facial expressions in their respective treatments. These results explore the potential multiple dynamics of facial expressions that were capable of distinguishing treatments in previous inferential analyses (modeling).

## Discussion

Heart rate (HR) and heart rate variability (HRV) have been extensively used to investigate the functioning of the autonomic nervous system (ANS), particularly the balance between vagal and sympathetic activity, in relation to coping strategies in horses.

The vagal branch of the ANS is associated with adaptive responsiveness to the environment [45], where individuals with higher parasympathetic activity tend to be more exploratory and adaptive to environmental demands.

In humans, the LF/HF ratio < 1.00 indicates a high level of relaxation of the body, characterizing parasympathetic activity on the heart [46], a fact also reported in a study by [47] who evaluated horses and found greater parasympathetic tone with values < 1.00. In Experiment I, the LF/HF ratio for mares presented values > 1.50 (ranging from 1.99 to 3.09), while in Experiment II, the ratio for geldings was > 1.00 (ranging from 1.06 to 2.98). Although there was no statistical difference between treatments, in both experiments, it was observed that the animals were under stress due to movement restriction from being restrained in the stock, with geldings showing greater resilience compared to mares. A study [48] found that sex may play an important role, with mares exhibiting a more pronounced stress reaction, showing higher values compared to geldings.

In investigating the reliability of heart rate variability (HRV) measures in horses [5], found that within a 4 m^2^ stall, where animals had the possibility of free, albeit limited or controlled, movements, parasympathetic control predominated. However, when restricted in a stock, activation of the sympathetic nervous system was observed, indicating that restriction of movement in the stock causes stress in the animals.

According to [29,49], the resting heart rate for adult horses, measured in environments with high temperatures (22°C to 36°C), can range from 28 to 40 beats per minute. In Experiment I, the heart rate of the mares was above the normal range for the equine species, showing values from 44 to 51 bpm. However, classical and country music were able to reduce the heart rate over time (p < 0.05), suggesting a calming effect.

A study conducted with women [50], indicated that the sympathetic nervous system is suppressed and the parasympathetic system is increased during exposure to classical music such as ‘Pachelbel’s Canon’ by the Jean-François Paillard Orchestra, suggesting a relaxing effect based on physiological parameters.

Contrary to the findings of [51], where country music induces agitation in various species due to its rhythm, the present study observed a relaxing effect. Additionally [52], conducted a study with humans and found that during auditory stimulation with heavy metal music, which is characterized by an agitated rhythm similar to country music, there was also a reduction in heart rate, indicating an acute decrease in autonomic cardiac regulation.

Consistent with the findings from Experiment I, musical genres such as country and classical positively affect the well-being of cattle, resulting in lower heart rates [53]. Additionally [54], reported that in everyday management situations for horses, classical music reduced the intensity of stress responses during short-duration transport or farrier treatments, suggesting that music may have practical implications.

In Experiment II, geldings showed heart rates ranging from 36 to 43 bpm, values close to the baseline range for the species, with no significant differences between treatments (p > 0.05). This contrasts with the results of [32], who exposed socially isolated male ponies to Hank Williams Jr.‘s country music and observed increased time spent feeding and a reduction in whinnying compared to the period without music.

The basal respiratory rate for horses in environments with high temperatures is between 10 and 20 breaths per minute [27,48]. In Experiments I and II, the results obtained were close to the established baseline range for the species, with values ranging from 19 to 28 and 16–25 breaths per minute, respectively. It is noteworthy that Pantaneiro horses are adapted and resilient to their environment, as reported by [28,29], who found that activities involving tourist rides and cattle handling in the Pantanal region did not significantly alter respiratory and heart rates before and after physical exertion.

The respiratory rate (RR) of geldings was lower at the initial time point (P1) of the control treatment, differing from the other treatments (p < 0.05). This effect may be related to the ‘novelty,’ meaning that the animals were exposed to unfamiliar auditory stimuli for the first time. However, although not significant, there was a trend towards an inversion in the responses where the RR of the control treatment increased numerically, while the RR of the musical genres decreased.

In Experiment I, mares exposed to classical music showed an increase in body temperature over time, suggesting a reduction in stress during restraint handling. A study with humans [49] also observed an increase in surface temperature of participants when listening to classical music, with a tendency for increased blood flow, indicating a potential calming effect.

Despite controversial responses regarding surface temperature and stress, the literature [55] supports this variable as an indicator of stress or well-being, where acute responses have been associated with patterns of vasoconstriction and vasodilation. In research conducted with healthy humans [56], found that stress led to changes in skin temperature, decreasing distally but increasing proximally, or remaining unchanged, indicating that there is no direct translation of this stress-induced hyperthermia paradigm.

The increase in the auricular temperature of mares exposed to classical music suggests relaxation or a lower level of stress. This result is consistent with reports from [56], which indicated that during a defensive/alarm response in horses, vasoconstriction can occur, leading to a subsequent decrease in auricular temperature. Similarly [57], observed a progressive reduction in the temperature of the auditory pavilion in rabbits subjected to a 15-minute immobilization procedure. Restraint is an action that induces fear and tension in animals regardless of species. Therefore, it can be inferred that the reduction in auricular temperature may be a plausible indicator of the potential calming effect of music on animals in stressful situations.

In Experiment II, the ocular temperature of the geldings was influenced (p < 0.05) by country music and the control (no music), showing a consistent increase over time, followed by a peak and subsequent decrease. This pattern of temperature elevation in response to a stressor is likely due to increased dilation of the ocular blood vessels and heightened visual attention [48].

Monitoring changes in ocular temperature has been used to assess equine responses to potentially stressful situations [58–61]. A decrease followed by an increase in temperature around the eyes (lacrimal caruncle) is associated with sympathetic [62] and parasympathetic [60,63] nervous system responses, respectively. In one study [64], observed an increase in ocular temperature in horses during potentially aversive handling, such as clipping, with temperature peaking after 10 minutes of the activity and decreasing immediately after the procedure. However, the ocular temperature did not return to baseline levels, possibly due to the metabolic consequences of the stress response.

To complement the physiological responses, the facial expressions of the animals were analyzed, focusing on those most indicative of stress, as evidenced by previous research on horses. In Experiment I, the facial expressions of *nostril dilator* (AD38), *ear rotator* (EAD104), and *half blink* (AU47) in mares exhibited significant treatment effects (p < 0.05). In Experiment II, with geldings, the treatment effect was significant only for the expression of *ears forward* (EAD101).

*Nostril dilator* (AD38) is a facial movement associated with deep breathing and sniffing, where the nostril diameter can vary depending on the animal’s physiological and psychological state [65]. Nostril dilator was observed in alert postures where the cardiorespiratory system is activated to prepare for potential flight [66], and it is detected in stressful situations [17]. Mares exhibited a higher frequency of this expression when exposed to new age music, concurrently with an increased frequency of *ear rotator* (EAD104).

In a study by [67], it was found that *ear rotator* (EAD104) is present in stressful situations resulting from social isolation. In Experiment I, the movement of the ear rotator, contrary to the direction from which the music was playing in front of the animal, suggests that during exposure to new age music, mares exhibited an aversion to the music. This expression occurred concurrently with an increased frequency of *half blink* (AU47).

*Half blink* (AU47) is characterized by a reduction in eye opening by the eyelids, without complete closure of the eyes. It is prevalent in horses in situations of pain [68], which can be considered a stressor [69]. In Experiment I, mares exhibited a higher frequency of half blink during the control treatment (no music), displaying a pattern similar to other stress-related facial expressions, suggesting that the animals were experiencing stress due to the restraint.

In contrast, *blink* (AU145) and *eye closure* (AU143) were more pronounced in mares during exposure to country and classical music, being the only expressions that exhibited behavior opposite to the others, suggesting an indication of relaxation. This might have contributed to the animals keeping their eyes closed for longer periods and/or more frequently, which is a requirement for coding both expressions, differentiated only by the speed of occurrence [15]. When analyzing the multiple dynamics of facial expressions in geldings, a similar trend was observed, with *blink* (AU145) showing behavior opposite to the others, being more evident during the new age treatment.

In Experiment II, geldings exhibited a higher frequency of *ears forward* (EAD101) during exposure to country music and a lower frequency when exposed to new age music. According to [70], the movement of ears forward occurs in horses under conditions of attention. This increase suggests that country music induced a state of alertness, with the animals keeping their ears directed forward for a larger portion of the time toward the auditory stimulus.

The results further indicate that in Experiment I, the lowest frequencies of facial expressions observed in mares—specifically *nostril dilator* (AD38), *ear rotator* (EAD104), and *half blink* (AU47)—occurred during exposure to classical and/or country music. These findings are consistent with the physiological parameters showing a reduction in average heart rate during classical and country music, and an increase in both auricular and body temperatures during classical music. This suggests a preference for classical and country music among the mares, which were able to reduce stress and provide a relaxing effect during restraint in the stocks. Conversely, the new age music, which had effects similar to the absence of music, was perceived as aversive.

This preference of mares for classical and country music genres can be explained by the frequency and tone characteristics of the music. As previously noted, the music used in the study had lower tones and frequencies around 15.000 Hz. The auditory sensitivity of horses is optimal between 1.000 Hz and 16.000 Hz [10]. A study by [71] aimed at identifying mare’s preferences for certain types of vocalizations found that they were more attracted to and showed greater attention to stallions with low-frequency vocalizations, indicating a potential biological preference.

The ‘Horsing Around’ music, of the new age genre, used in the experiments was specially produced for horses. However, the divergent results observed in Experiment I with the mares were similar to those found in another study by [72], which investigated the effect of music on stress reduction in dogs and also found no benefits with music specifically created for that species.

In contrast, in Experiment II, the facial expressions of the geldings suggest a possible preference for the new age music genre, corroborating a study that evaluated the same genre of music for geriatric horses and confirmed its positive effect on relaxation [11]. Classical and country music showed a similar effect to the control treatment. This is evidenced by the increase in ocular temperature observed during both the country music and control treatment, indicating a stress response. Thus, it can be inferred that the geldings reacted indifferently to the music, with no effective reduction in stress.

Notably, there is a difference between mares and geldings regarding preferences, including musical genres. Differences have also been observed in humans, where [73] suggested that psychophysiological responses to music may be influenced by hormonal state. In this context [74], indicated that men are more likely to experience less anxiety than women when exposed to a stressor, suggesting that they may become less anxious when the stressor is less imminent. This phenomenon is also observed between geldings and mares [75].

Similarly, this justification can be applied to the differences in skin temperature and autonomic cardiac modulation. [51] described that in a small sample of male volunteers, there was no influence of musical auditory stimulation with varying times and styles.

The results of this study underscore the complexity of stress responses in horses and the necessity for a personalized approach to animal welfare management. Factors such as the genre of music, the sex of the animals, and specific handling conditions should be carefully considered to maximize the benefits of auditory enrichment. Classical and country music proved promising in reducing stress in mares, while geldings may respond more positively to new age music. For practical application of these findings to improve the welfare of horses in controlled environments, it is essential to consider the genre of music, sex, breed, handling, and duration of exposure.

## Conclusions

The ‘9th Symphony’ by Beethoven (classical genre) and ‘Ramblin’ In My Shoes’ by Hank Williams Jr. (country genre) promote stress reduction and a consequent relaxing effect in Pantaneiro mares during social isolation and movement restriction. In contrast, Pantaneiro geldings exhibited a less pronounced response, showing a possible preference for ‘Horsing Around’ by Janet Marlow (new age genre) under the same handling conditions.
